# The Endogenous Metabolite Glycerophosphocholine Promotes Longevity and Fitness in *Caenorhabditis elegans*

**DOI:** 10.3390/metabo12020177

**Published:** 2022-02-14

**Authors:** Jia-Yu Liu, Run-Qi Zheng, Yao Wang, Yan-Hong Liu, Shuai Jiang, Xin-Zheng Wang, Kun He, Xin Pan, Tao Zhou, Tao Li, Qing Xia, Wei-Na Zhang

**Affiliations:** 1 State Key Laboratory of Proteomics, National Center of Biomedical Analysis, Beijing 100850, China; liujiayu6066@163.com (J.-Y.L.); rqzheng@xmail.ncba.ac.cn (R.-Q.Z.); ywang@xmail.ncba.ac.cn (Y.W.); yhliu@xmail.ncba.ac.cn (Y.-H.L.); sjiang@xmail.ncba.ac.cn (S.J.); wxz@proteomics.cn (X.-Z.W.); hk@proteomics.cn (K.H.); xpan@ncba.ac.cn (X.P.); tzhou@ncba.ac.cn (T.Z.); tli@ncba.ac.cn (T.L.); 2 Nanhu Laboratory, Jiaxing 314000, China

**Keywords:** *Caenorhabditis elegans*, glycerophosphocholine, lifespan, healthspan, stress resistance

## Abstract

Metabolism and aging are closely connected. The choline derivative glycerophosphocholine (GPC), an important precursor of the neurotransmitter acetylcholine, plays important roles in brain and nervous system function. Although it has been reported to alleviate cognitive decline in aged mice, whether GPC could promote longevity and other fitness factors remains unclear. Here, we find endogenous GPC level declines in the plasma of ageing humans. In *Caenorhabditis elegans* (*C. elegans*), GPC extends lifespan and improves exercise capacity during aging. Likewise, GPC inhibits lipofuscin accumulation. We further show that GPC treatment has no adverse effect on nematodes’ reproductive abilities and body length. In addition to its benefits under normal conditions, GPC enhances the stress resistance of *C. elegans*. Mechanically, we find GPC significantly inhibits the reactive oxygen species (ROS) accumulation in worms. Our findings indicate the health benefits of GPC and its potential application in strategies to improve lifespan and healthspan.

## 1. Introduction

Aging, a universal physiological phenomenon, is comprehensive manifestation of the decline and disorder in both structural homeostasis and functional integrity, gradually resulting in health impairment [1]. As an irreversible biological process, aging serves as a pathogenic factor of various chronic diseases, such as neurodegenerative diseases, cardiovascular diseases, and so on [2]. In recent years, exploring for novel anti-aging molecules has become one of the most fascinating biology research topics. Many aging regulators have been identified. Dr. Wes Collet et al. reported the upregulation of plasma proteins CCL11 and VCAM1 during aging. They showed that increasing peripheral CCL11 in young mice damaged learning and memory ability [3], while VCAM1 inhibition reversed the cognitive impairment of ageing mice [4]. Facing the increase in the ageing population, it is of great significance to search for anti-aging strategies to improve the quality of life of the elderly.

Endogenous metabolites participate in important physiological and pathological processes of the body. Due to their natural, safe and potentially medicinal properties, endogenous metabolites have attracted more and more attention. Several metabolites have recently shown anti-aging activity. Studies have reported that the intermediate product of the tricarboxylic acid cycle, α-ketoglutaric acid, and the crucial coenzyme nicotinamide adenine dinucleotide (NAD+) promote healthspan and lifespan in *C. elegans* and mouse models [5,6,7,8]. Furthermore, the supplement of N-glycan precursor N-acetylglucosamine could extend lifespan in *C. elegans*, and alleviate the pathology of several distinct neurotoxic disease models [9]. 

Glycerophosphocholine (GPC) is a water-soluble choline molecule that is included in foods such as milk and soy [10]. It is known to be the biosynthetic precursor of neurotransmitter acetylcholine. In humans, glycerophosphocholine phosphodiesterase (GPCPD1) is the key enzyme converting GPC to choline, and choline o-acetyltransferase (CHAT) is responsible for converting choline to acetylcholine. In *C. elegans*, *gpcp-1* and *gpcp-2* are the ortholog genes of GPCPD1, and *cha-1* is the ortholog gene for CHAT. Dr Jamuna R. Subramaniam’s group reported that Reserpine, an FDA-approved antihypertensive drug, could modulate neurotransmitter release to extend lifespan and alleviate age-dependent Aβ proteotoxicity in *C. elegans*. They further identified acetylcholine as the crucial player in reserpine’s action, since reserpine could not extend lifespan in *C. elegans* without *cha-1* expression [11]. The literature has also shown some relief effect of GPC on senescence, transthyretin deposition, and osteoarthritis in aged mice [12]. The intake of GPC not only prevents the decline of taste sensitivity and energy regulation in aged mice, but also protects against cognitive decline in patients with Alzheimer’s disease [13,14]. Although GPC has been reported to alleviate age-related symptoms, it is not clear whether it can improve lifespan and healthspan, including mobility fitness.

*C. elegans* is recognized as a classic biological model for aging research due to its short life cycle, genetic traceability and tractability [15]. Nearly 80% of genes of *C. elegans* are homologous with human genes [16]. In this study, by using *C. elegans* as a model organism, we have found that the endogenous metabolite GPC promotes lifespan and fitness during aging. Moreover, the molecular mechanisms underlying GPC’s ability to extend lifespan are elucidated. These results enhance our understanding of GPC’s benefits for healthspan and lifespan.

## 2. Results

### 2.1. Endogenous Metabolite GPC Prolongs the Lifespan and Improves the Fitness in C. elegans 

**Figure 1:**
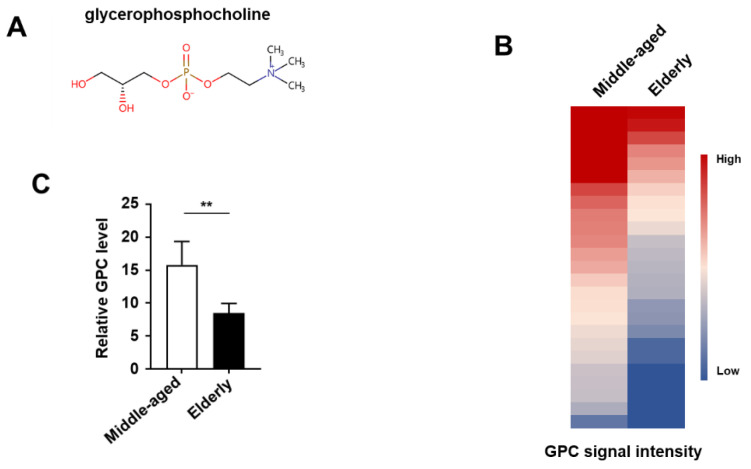
Plasma GPC level declines during aging. (**A**) Scheme of chemical structure for GPC. (**B**) Heat-map analysis of GPC signal intensity in plasma samples from middle-aged (*n* = 25) and elderly people (*n* = 25). Each column in the heat map represents one age group, and each row represents one sample from the middle-aged or elderly group. Plasma GPC level was detected by UPLC-ESI-MS/MS. (**C**) The relative abundance of plasma GPC in each group. ** *p* ≤ 0.01; by unpaired t test. Values are mean ± s.e.m.

To investigate whether endogenous metabolites participate in aging regulation, we employed an unbiased, systemic metabolomics approach to examine the global metabolic changes during aging. Plasma samples from 25 middle-aged people (mean age 45.1 ± 0.74 years) and 25 elderly people (mean age 71.4 ± 6.81 years) were collected and non-targeted metabolomics analysis was performed. Among the identified metabolites that changed with age, we found that the plasma GPC level decreased in elderly people as compared to younger ones (Figure 1A–C). 

**Figure 2:**
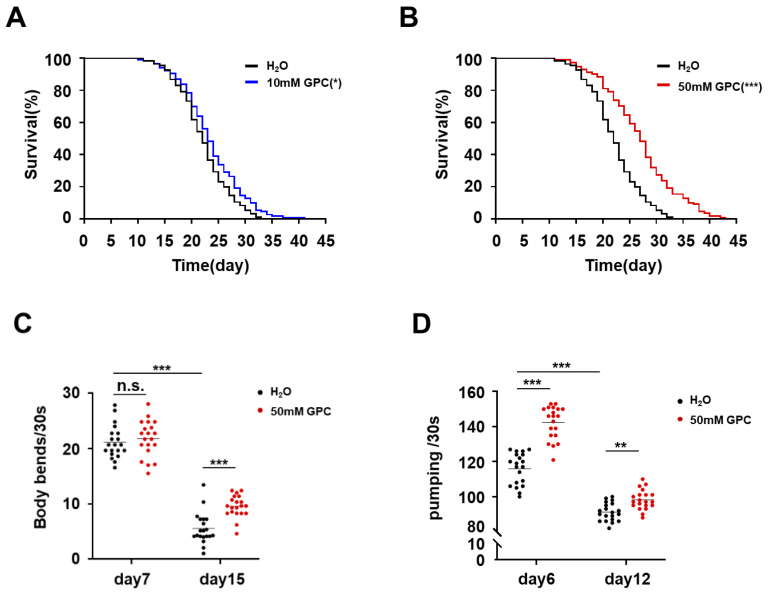
GPC extends lifespan and improves fitness of *C. elegans*. (**A**,**B**) Lifespan of worms treated with GPC (10, 50 mM) or vehicle (H_2_O). *p* values represent comparison with vehicle calculated using long rank test. (**C**) Body bending frequency on days 7 and 15 of adulthood in worms treated with 50 mM GPC or vehicle (*n* = 20). (**D**) Pharyngeal pumping frequency on days 6 and 12 of adulthood in worms treated with 50 mM GPC or vehicle (*n* = 20). For (**C**,**D**), ** *p* ≤ 0.01; *** *p* ≤ 0.0001; n.s., not significant; by two-way analysis of variance (ANOVA) followed by Bonferroni tests. Values are mean ± s.e.m.

To investigate the anti-aging effect of GPC, we first evaluated the effect of GPC on lifespan by using *C. elegans*. Compared to the control group, a supplement of 10 mM GPC had a slight effect on whole lifespan, mean lifespan, as well as maximum lifespan (Figure 2A; Table 1). However, the treatment of nematodes with GPC at a final concentration of 50 mM obviously extended the lifespan of *C. elegans* (Figure 2B). Meanwhile, a 50 mM GPC treatment also led to an obvious extension of mean lifespan and maximum lifespan (Table 1). It is well-known that the motor ability decreases with age [17,18].Therefore, we next examined the effect of GPC on worm activity by evaluating the mobility and pharyngeal pumping rate. We scored nematodes’ activity by counting body bending rate and pharyngeal pumping rate at different stages of life cycle. As shown in Figure 2C, D, both body bending frequency and pharyngeal pumping rate decreased during aging. Notably, compared with vehicle, the treatment with 50 mM GPC robustly improved both activities. GPC enhanced the motor activity of body bends at the mid-late period of life stage, whereas it increased the frequency of pharyngeal pumping at the early and mid-life stages. These results indicate that GPC alleviates the decline in body bending frequency and pharyngeal pumping rate during aging. Taken together, these data suggest that the metabolite GPC significantly extends the lifespan and promotes fitness in *C. elegans.*

### 2.2. GPC Inhibits the Lipofuscin Accumulation in C. elegans

**Figure 3:**
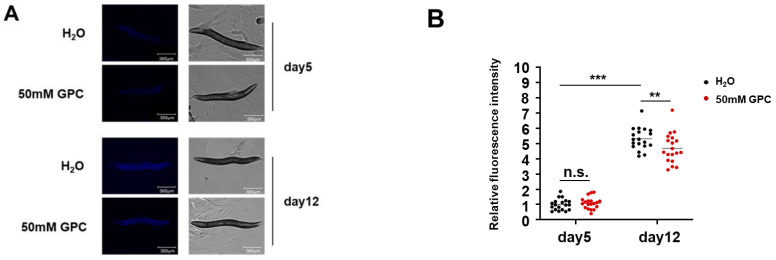
GPC reduces the lipofuscin accumulation in *C. elegans*. (**A**) Representative intestinal autofluorescence and brightfield images (*n* = 19 images per group) of worms on days 5 and 12 of adulthood after being treated with 50 mM GPC or vehicle. The blue autofluorescence in the figure indicates the accumulation of lipofuscin. Scale bars, 300 µm. (**B**) The comparison of relative fluorescence intensity of intestinal autofluorescence in groups as indicated in A. ** *p* ≤ 0.01; *** *p* ≤ 0.0001; n.s., not significant; by two-way analysis of variance (ANOVA) followed by Bonferroni tests. Values are mean ± s.e.m.

Lipofuscin is a pigment that fluoresces automatically in the gut of *C. elegans*. It cannot be eliminated by exocytosis and accumulates in cells in an age-dependent manner, thus reflecting the rate of aging and health status in nematodes [19]. Since the level of intestinal lipofuscin is an important marker during aging [20], we evaluated the effects of GPC on the deposition of intestinal lipofuscin on days 5 and 12 of adulthood. We demonstrated that the lipofuscin fluorescence accumulated with age, while GPC treatment robustly prevented the accumulation of relative fluorescence intensity at the mid-late stage (day 12) (Figure 3A,B). These data support our conclusion that GPC significantly delays the ageing process in *C. elegans*.

### 2.3. GPC has no Adverse Effect on the Fertility and Body Length of C. elegans

**Figure 4:**
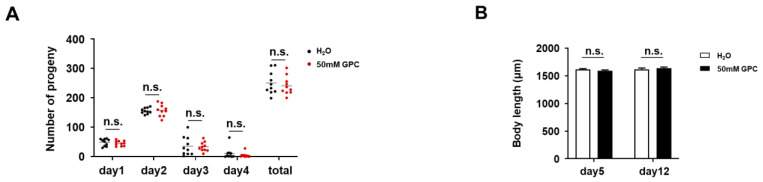
GPC has no adverse effect on the fecundity and body length of *C. elegans*. (**A**) The number of progenies on days 1, 2, 3 and 4 was scored, and the total number of progenies was summarized. (**B**) The body length of nematodes on days 5 and 12 was measured. n.s., not significant; by two-way analysis of variance (ANOVA) followed by Bonferroni tests. Values are mean ± s.e.m.

Lifespan extension is often related to reductions in or losses of reproductive ability [21,22]. To test whether GPC impairs fertility in *C. elegans*, we performed a fecundity assay. The number of offspring on the first 4 days of reproductive time was determined and the total progeny production was recorded. As Figure 4A showed, both the daily and total numbers of progenies over the 4 days were barely affected by GPC treatment. These data indicate that 50 mM GPC treatment does not threaten reproductive capacity. Thus, the lifespan extension seemed not to be associated with the loss of fertility. In addition, we found that the body length of nematodes with GPC supplement on days 5 and 12 stayed the same as those in the control group (Figure 3A and Figure 4B). These results suggest that GPC could significantly enhance the healthspan of nematodes without affecting their fertility and length.

### 2.4. Glycerophosphocholine Enhances Stress Resistance in C. elegans

**Figure 5:**
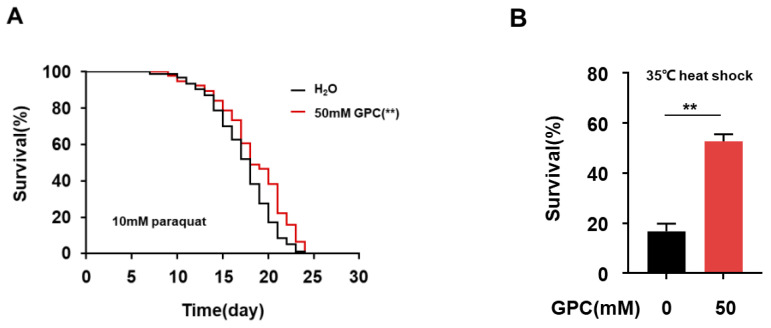
GPC enhances stress resistance in *C. elegans*. (**A**) Representative survival curves of worms under oxidative stress. The nematodes on day 6 of adulthood treated with or without 50 mM GPC were transferred to the NGM plate with 10 mM paraquat. *p* values represent comparison with vehicle calculated using long rank test. (**B**) Survival for day 2 nematodes treated with or without 50 mM GPC was recorded after being exposed to 35 °C heat shock for 7 h. ** *p* ≤ 0.01 by unpaired t test. Values are mean ± s.e.m.

Previous studies have shown that the improvement of stress resistance contributes to lifespan extension in *C. elegans* [23,24]. To investigate the potential protective effect of GPC under oxidative stress, we transferred the day 6 adult nematodes to an NGM plate with 10 mM paraquat, a toxic agent known to continuously generate oxidative stress emanating from the mitochondria. The survival curve demonstrated a statistically significant protective effect of GPC at 50 mM in comparison to the control group (Figure 5A). As Table 2 showed, GPC treatment significantly prolonged the mean lifespan and maximum lifespan of *C. elegans* upon paraquat-induced oxidative stress. Similarly, GPC treatment improved stress resistance induced by heat shock in *C. elegans*. As shown in Figure 5B, after culturing under 35 °C heat shock conditions for 7 h, GPC-treated nematodes exhibited significantly higher survival rates than the control group. These data demonstrate that GPC strengthens resistance capacity upon oxidative stress and heat shock in *C. elegans*.

### 2.5. GPC Decreases the Intracellular ROS Level in C. elegans 

**Figure 6:**
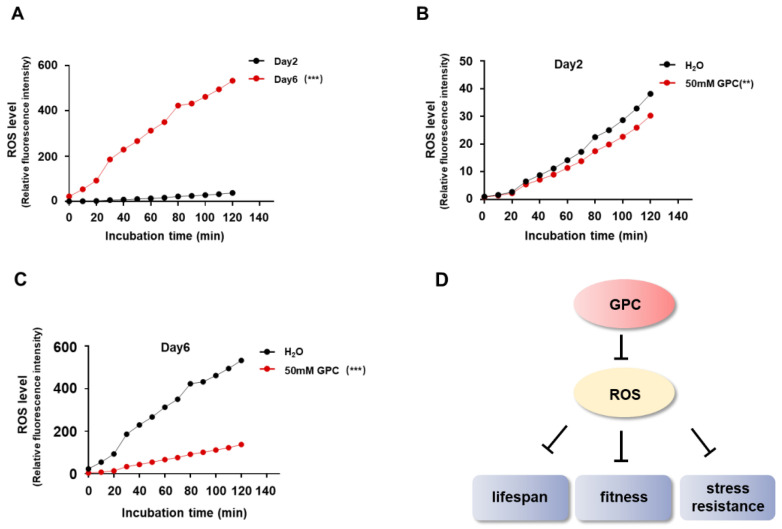
GPC reduces ROS accumulation in *C. elegans* with age. (**A**) The level of intracellular ROS increased during aging. In total, 500 nematodes on day 2 and day 6 of adulthood were harvested and measured for ROS levels with the fluorescent dye DCF. The fluorescence intensity was measured every 10 min for 2 h at 37 °C. (**B**,**C**) GPC inhibited ROS accumulation in *C. elegans* on day 2 (**B**) or day 6 (**C**). Nematodes on day 2 or day 6 of adulthood treated with or without GPC were harvested and ROS level was detected as in (**A**). (**D**) Illustration of GPC function in promoting lifespan and healthspan. ** *p* < 0.001; *** *p* < 0.0001; by two-way analysis of variance (ANOVA) followed by Bonferroni tests. Values are mean ± s.e.m.

The free radical theory indicates that cells and organisms could be damaged by the intracellular ROS [25], and the lifespan of nematodes is negatively correlated with ROS level [26,27]. To gain insights into how GPC prolongs lifespan and enhances the stress resistance in *C. elegans*, we used H2DCF-DA as an indicator to monitor the intracellular amount of ROS in *C. elegans*. As shown in Figure 6A, the ROS accumulation of day 6 worms was significantly higher than that of day 2 worms. Notably, GPC robustly inhibited the ROS accumulation at different life stages (Figure 6B,C). These results suggest that GPC reduces intracellular ROS accumulation with age, which might contribute to the improvement of longevity, fitness and stress tolerance (Figure 6D). 

## 3. Discussion

Discovering longevity regulators that delay aging and extend lifespan has long been a dream for human beings. Much of the literature has shown that series of molecules participate in the regulation of the ageing process. The sirtuin family members are well known to modulate lifespan and healthspan through diverse mechanisms [7]. Dr. Andrew Dillin’s lab reported that hyaluronidase TMEM2 promoted ER homeostasis and extended longevity in *C. elegans* [28]. Moreover, Dr. Tony Wyss-Coray’s group identified several factors that affected brain aging and cognitive function in the mouse model, including a canonical B cell receptor, CD22 [29], the MHC molecule β2-microglobulin (B2M) [30] and so on. Until now, most of the molecules reported to regulate aging have been focused on various proteins or genes. Since metabolites play decisive roles in the control of complex mammalian cell systems, the involvement of endogenous metabolites in aging regulation has been widely acknowledged recently. Even though a few metabolites, such as α -ketoglutaric acid and nicotinamide adenine dinucleotide (NAD+)-related molecules, have shown obvious anti-aging effects in *C. elegans* and mouse models [5,7,8], other metabolites that benefit longevity or fitness remain to be discovered. In this study, we identified the metabolite GPC as a novel factor that promoted longevity and fitness in *C. elegans*. 

The choline derivative GPC is rich in foods such as milk and soybeans. As the biosynthetic precursor of acetylcholine, GPC has been reported to promote memory and learning capacity, and improve brain-transduction mechanisms [14]. Recent studies have begun to reveal the effect of GPC on aging-related disorders. Matsubara K. et al. reported the beneficial effects of GPC on senescence, transthyretin deposition, and osteoarthritis in senescence-accelerated mouse prone 8 (SAMP8) mice [12]. Evidence has shown that the GPC level declined in the hippocampus and cortices of aged rats compared to young and middle-aged groups [31]. The long-term feeding of GPC could prevent aging-related cognitive decline in old C57BL/6J mice [14]. Notably, Thomas J. Wang’s lab reported that plasma GPC level was closely associated with attaining longevity in humans [32]. Before now, whether GPC could promote longevity and other fitness factors has not been clear. In the present study, we found that the endogenous metabolite GPC declined in plasma with age, indicating its possible role in longevity regulation. By using the *C. elegans* model, we first investigated the effect of GPC on lifespan as well as excise activity during aging. We showed the beneficial effect of GPC not only on normal lifespan, but also on stress-conditioned survival. Thus, our study revealed the novel function of GPC in extending lifespan and promoting healthspan.

As is well known, ROS accumulates progressively with age, which is an important causative factor for aging. Our work has demonstrated that GPC reduced ROS accumulation during aging. These findings suggest a model in which GPC is a key metabolite mediating lifespan extension by reducing ROS accumulation. The detailed mechanisms underlying GPC affecting ROS level need to be further investigated. In addition, there is another possible longevity mechanism called “trade-off”, in which longevity promotion could be obtained at the expense of losing reproductive ability [21]. However, based on our results, GPC improved lifespan and fitness without sacrificing fertility in *C. elegans*, making it a safe longevity compound.

Longevity molecules that delay aging and extend lifespan have long been explored. In the present study, we show that a natural metabolite, GPC, extends lifespan and improves fitness in *C. elegans*, which provides new strategies to manipulate the ageing process via the restoration of metabolism homeostasis. Whether these health benefits observed in *C. elegans* can be replicated in mammals remains to be explored. 

## 4. Materials and Methods

### 4.1. Reagents

The wild-type (N2) *C. elegans* strain and *Escherichia coli* OP50 (*E.coli* OP50) were supplied by the Hong Zhang laboratory, University of Chinese Academy of Sciences (Beijing, China). 5-Fluoro-2′-deoxyuridine (FUDR), 2′,7′-dichlorofluorescin diacetate (DCFH-DA) and 5-Methyltryptophan (paraquat) were purchased from Sigma-Aldrich, Co. (St. Louis, MO, USA). GPC was purchased from Beijing Energy Engineering Technologies Co., Ltd. (Beijing, China). 

### 4.2. Metabolite Extraction from Plasma 

Plasma samples were collected from fifty healthy subjects. The volunteers were divided into two groups, including 25 middle-aged people (40–45 years old) and 25 elder people (63–83 years old). The study was approved by the local institutional ethics committee, and informed consent was obtained from all study participants. Blood from healthy subjects were centrifuged and plasmas were collected for metabolites extraction. Plasma samples were mixed with precipitant, and then placed at −20 °C for 1 h. Samples were centrifuged (12,000 rpm, 4 °C) for 15 min, and the supernatants were frozen at −80 °C before UPLC-ESI-MS/MS analysis.

### 4.3. UPLC-ESI-MS/MS Analysis

The metabolomic analysis was performed as described previously [33]. Briefly, the metabolite profiling of all samples was acquired by an ultrahigh-performance liquid chromatography system coupled to a hybrid Q-TOF mass spectrometer (UPLC-QTOF-MS) in both positive and negative ion modes. The mobile phases were water with 0.1% FA and acetonitrile with 0.1% FA. Three technical replicates (three injections) were performed for each sample in the four analysis modes.

### 4.4. C. elegans Maintenance

The nematodes were raised and maintained at 20 °C in 60 mm dishes containing nematode growth media (NGM) and seeded with *E. coli* OP50. Gravid hermaphrodites were treated with lysis solution containing 10 M NaOH and 5% NaOCl. About three days after synchronization, the progenies reached young adulthood and then were transferred to the test dishes for further experiments. 

### 4.5. Lifespan Assay

Lifespan assays were performed according to the protocol described previously [34]. GPC stock solution (500 mM in water solution, stored at 4 °C) was mixed with *E.coli* OP50 to the final concentrations of 10 and 50 mM, respectively. About 100 synchronized nematodes in the L4 stage were used for the experiment. In total, 5 μM of FUDR was added into the NGM plates to prevent progeny production for 10 days, and then the worms were transferred onto ordinary NGM plates without FUDR. To prevent pollution and avoid a lack of food, the worms were transferred to a fresh plate every three days. Nematodes were counted every day until all were dead. Nematodes were scored as dead if they no longer responded to gentle stimulus with a platinum wire.

### 4.6. Locomotion Behavior Assay and Pharyngeal Pumping Assay

The motor ability of nematodes was evaluated by body bending frequency at different phases of the lifecycle. All the nematodes were treated with vehicle or 50 mM GPC from L4-larvae. To calculate the frequencies of body bends, day 7 and day 15 adulthood nematodes were placed onto new NGM plates without seeding *E. coli* OP50 and allowed to recover for 1 min. Subsequently, the frequencies of body bends were calculated for 30 s. Body bends were defined as a wavelength along the long axis of the body [35]. About 20 nematodes were counted in each group.

For the pharyngeal pumping assay, the nematodes were pretreated as described in Section 4.4. The frequencies of pharyngeal pumping were conducted on day 6 and day 12 of adulthood and counted on the plates directly. The number of pumps was recorded for 30 s with 20 nematodes in each group [36]. 

### 4.7. Lipofuscin Accumulation Assay

The intestinal lipofuscin deposition determination was performed as Wilson, M.A. et al. described [37]. On days 5 and 12 of adult life, nematodes were anesthetized by 10 mM levamisole hydrochloride and fixed on a 2% agarose plate. The intestinal fluorescence was visualized by a fluorescence microscope (Thermo EVSO5000, Thermo Fisher Scientific, Waltham, MA, USA) with ×10 magnification. Lipofuscin levels were quantified by determining average pixel intensity using ImageJ software in each worm’s intestine. More than 15 worms were examined in each group.

### 4.8. Fecundity Assay

For the fecundity assay, the NGM plates were treated with vehicle or 50 mM GPC without FUDR. The nematode of the L4 larvae was shifted to a fresh NGM plate every 24 h after laying eggs. Only one nematode on each plate and approximately ten worms were examined. This process was repeated continuously for the first four days of the life cycle. After culturing at 20 °C for 3 days, the daily number of progenies was counted, and then the total number of progenies was scored [38].

### 4.9. Body Length Assay

The nematodes supplemented with vehicle or 50 mM GPC were transferred onto the NGM plates at the L4 larval stage and cultured at 20 °C. On days 5 and 12 of life, nematodes were anesthetized by 10 mM levamisole hydrochloride and fixed on a 2% agarose plate. The nematodes were visualized using a bright-field microscope (Thermo EVSO5000, Thermo Fisher Scientific, Waltham, MA, USA) with ×10 magnification. The body length was measured using ImageJ software. More than 15 worms were examined in each group.

### 4.10. Stress Assays

For the oxidative stress assays, the 6-day-old nematodes from the control or GPC-treated group were shifted to the NGM plate containing 10 mM paraquat. The survival of 100 nematodes was recorded every day. For the heat stress assay, the nematodes with or without 50 mM GPC treatment were incubated at 35 °C on day 2 (*n* = 100) and the survival of nematodes was recorded every 1 h until all the nematodes were dead.

### 4.11. Determination of ROS in C. elegans

DCFH-DA, a fluorescent probe, was used to measure the intracellular level of ROS as described previously [39]. About 500 nematodes were collected with M9 buffer and washed 3 times. After centrifuging at 3000 RPM for 3 min, 500 μL of saline was to each tube. Ultrasound was then performed for 20 min until the worms disappeared. After centrifuging, the supernatant was collected and the precipitate was discarded. Subsequently, 50 μL supernatant was mixed with 50 μL DCFH-DA before adding into a 96-well plate. The fluorescence was detected by a fluorescence microplate reader (Molecular Devices, San Jose, CA, USA) with the excitation wavelength 485 nm and the emission wavelength 538 nm every 10 min for 2 h at 37 °C.

### 4.12. Statistical Analysis

All the experiments were repeated at least three times. Data were presented as the mean ± s.e.m. in this study. *p* values of survival curves represent comparisons with vehicle calculated using long rank test. The results were assessed for whether they differed significantly (*p* < 0.05) via two-way analysis of variance (ANOVA) followed by Bonferroni tests or unpaired t test using GraphPad Prism.

## Figures and Tables

**Table 1:** Effect of GPC on the lifespan of *C. elegans*.

Treatment	Total Number of Nematodes	Mean Lifespan (Days)	Maximum Lifespan (Days)	Mean Fold Increase (%)
H_2_O	98	22.02 ± 4.83	30.70 ± 1.27	
10 mM GPC	113	23.47 ± 5.67 *	34.00 ± 2.70 **	6.58
50 mM GPC	110	26.96 ± 6.71 ***	39.18 ± 1.80 ***	22.43

* *p* ≤ 0.05; ** *p* ≤ 0.01 *** *p* ≤ 0.0001 compared with control group.

**Table 2:** Effect of GPC on the oxidative stress resistance in *C. elegans*.

Treatment	Total Number of Nematodes	Mean Lifespan (Days)	Maximum Lifespan (Days)	Mean Fold Increase (%)
H_2_O	94	17.36 ± 3.30	22.56 ± 0.83	
50 mM GPC	90	18.46 ± 3.88 *	23.67 ± 0.47 **	6.34

* *p* ≤ 0.05; ** *p* ≤ 0.01 compared with control group.

## Data Availability

All data are provided in the manuscript.
